# Immunity to Visceral Leishmaniasis Using Genetically Defined Live-Attenuated Parasites

**DOI:** 10.1155/2012/631460

**Published:** 2011-09-06

**Authors:** Angamuthu Selvapandiyan, Ranadhir Dey, Sreenivas Gannavaram, Ines Lakhal-Naouar, Robert Duncan, Poonam Salotra, Hira L. Nakhasi

**Affiliations:** ^1^Institute of Molecular Medicine, 254 Okhla Industrial Estate, Phase III, New Delhi 110020, India; ^2^Division of Emerging and Transfusion Transmitted Diseases, Office of Blood Research and Review, Center for Biologics Evaluation and Research, Food and Drug Administration, Bethesda, MD 20892, USA; ^3^Institute of Pathology (Indian Council of Medical Research), Safdarjung Hospital, New Delhi 110029, USA

## Abstract

Leishmaniasis is a protozoan parasitic disease endemic to the tropical and subtropical regions of the world, with three major clinical forms, self-healing cutaneous leishmaniasis (CL), mucocutaneous leishmaniasis (MCL), and visceral leishmaniasis (VL). Drug treatments are expensive and often result in the development of drug resistance. No vaccine is available against leishmaniasis. Subunit *Leishmania* vaccine immunization in animal models has shown some efficacy but little or none in humans. However, individuals who recover from natural infection are protected from reinfection and develop life-long protection, suggesting that infection may be a prerequisite for immunological memory. Thus, genetically altered live-attenuated parasites with controlled infectivity could achieve such memory. In this paper, we discuss development and characteristics of genetically altered, live-attenuated *Leishmania donovani* parasites and their possible use as vaccine candidates against VL. In addition, we discuss the challenges and other considerations in the use of live-attenuated parasites.

## 1. Introduction

 Leishmaniasis is caused by protozoan parasites of the genus *Leishmania* of the family *Trypanosomatidae *and is transmitted by the sand fly vector. It infects about 12 million individuals globally in tropical and subtropical regions, with ~2 million new clinical cases (0.5 million visceral leishmaniasis (VL) and 1.5 million cutaneous leishmaniasis (CL)) reported annually with an estimated death toll of ~50,000 persons/year [1]. The three major clinical forms of leishmaniasis, VL, CL, and MCL are the result of infection by different species of the parasite and the immune response of the host. VL, fatal if not treated, is caused by *L. donovani, L. infantum, *and *L. chagasi * [2, 3]. More than 90% of the visceral cases in the world are reported from Bangladesh, Brazil, India, and Sudan. Most affected patients (70%) are children under 15 years of age who already suffer from concurrent malnutrition and other secondary illnesses. The major clinical symptoms for VL are characterized by prolonged and irregular fever, splenomegaly, and hepatomegaly [3]. CL causes lesions that are self-healing and are caused by *L. major, L. tropica,* or *L. aethiopica* in the old world and by *L. mexicana* or *L. braziliensis* complex in the new world [4]. MCL is potentially life threatening and affects the mucosal region of infected individuals, typically seen in the Central and South America and caused by *L. braziliensis, L. amazonensis, L. panamensis, and L. guyanensis *[3].

In the *Leishmania *life cycle, motile promastigote forms that reside in the gut of the sand fly vector are transmitted to a mammalian host during a blood meal. Parasites are taken up by the neutrophils that are ingested by the host macrophages, differentiate into the nonmotile amastigote form, and reside and multiply in the phagolysosome compartment of the macrophages. These two major life stages have been adapted to *in vitro *culture for most *Leishmania *species allowing manipulation of the genome and assessment of the altered phenotypes *in vitro *and* in vivo*. 

The most significant public health effects of leishmaniasis are concentrated in developing countries. Dogs represent an important reservoir for the parasite in Europe, the Middle East, as well as Latin America. In the USA, even though leishmaniasis is not endemic, infections can be observed in pockets of the country, mainly in the southwest [5, 6]. In addition, VL due to *L. infantum *was found in fox hounds in the northeastern part of the USA and transmission may involve exposure to an insect vector, direct transmission, or vertical transmission [7]. In addition, there have been several documented cases of parasite transmission by blood transfusion globally [8] and an outbreak of leishmaniasis among US troops led to deferral from donating blood for travel to Iraq in 2003 [9]. Studies in animal models, such as hamsters and dogs show that *Leishmania* not only survives blood-banking storage conditions, but also retains its infectivity [10, 11]. This retention of infectivity in blood raises the possibility of spread of disease from asymptomatic individuals through transfusion to healthy individuals in areas where the sand fly vector is absent. 

Currently, the only treatment for leishmaniasis is drug treatment. However, either prolonged use or inefficient drug therapy has resulted in drug resistance. Currently, more than 60% of the clinical cases are resistant to the first line drug, antimony [12]. There is concern about the possibility of development of resistance to the new oral drug miltefosine in the near future as there are already reports of relapse among miltefosine treated cases [13]. In addition, about 50% of Sudanese and 5–10% of Indian kala azar patients within months after successful treatment develop post-kala-azar dermal leishmaniasis (PKDL) [14]. PKDL is a condition characterized by the appearance of diffuse lesions in the skin that harbor the visceral parasite and is considered a reservoir for transmission in these countries. There are no vaccines available at present. Attempts to develop vaccines for such parasitic agents such as heat killed, subunit, or DNA vaccines have not resulted in a successful vaccine candidate that could be applicable to humans [15–17]. However, past experience has revealed that individuals who recover from *Leishmania* infection develop a long lasting protection from future infections suggesting that a successful vaccine candidate needs to cause a controlled infection that evokes a protective immunity. Experience from other pathogens has suggested that a live-attenuated parasite vaccine could fulfill such requirements [18, 19]. Hence, in this paper we have focused on current knowledge about the (a) immunology of leishmaniasis, (b) need for and development of live-attenuated *Leishmania* parasites as vaccine candidates, (c) the safety challenges such parasites present, (d) the type of immune responses generated by such parasites that are protective, (e) experimental animal models for testing vaccines, and (f) routes of administration and mode of virulent challenge for vaccinated animals. Recently, various review articles have been published discussing the role of genetically altered *Leishmania* as vaccine candidates [3, 16, 17, 20–23]. In this paper we have limited our scope to genetically altered *L. donovani* parasites and their use as vaccine candidates.

## 2. Immunology of Leishmaniasis

 Immunity to leishmaniasis is mediated by both arms of mammalian cellular immune system; innate (by neutrophils, macrophages, and dendritic cells) and adaptive (T cells) responses [20]. The sand fly bite causes minimal tissue damage that promotes recruitment of neutrophils to the site of injury as a primary immune defense mechanism of the host [24, 25]. However, regardless of *Leishmania* species, these neutrophils are the primary target of this parasite. Despite the harsh intracellular environment, parasites can survive for a time period, and eventually these parasitized neutrophils or viable parasites are engulfed by macrophages or dendritic cells. *Leishmania* can survive and replicate inside macrophages by modulating the normal antimicrobial machinery as well as increasing the host cell membrane fluidity and disrupting lipid rafts, which in turn affects the antigen presentation capability of host APCs [26]. These parasitized APCs then interact with T cells to stimulate cytokines, and in this whole process, several cytokines and chemokines are involved. In this way, *Leishmania* starts to hijack the whole immune system for its survival [27]. 

In leishmaniasis, host defense against intracellular *Leishmania* is cell mediated, which involves Th1 responses due to T-cells primed primarily by dendritic and macrophage cells producing IL-12 [28–31]. A clear dichotomy between Th1-mediated protection (mediated by major cytokines IFN*γ*, IL-2, TNF) and Th2-mediated disease progression (mediated by major cytokines IL-10, IL-4) has been demonstrated in mice against cutaneous leishmaniasis [21, 32]. However, this Th1/Th2 dichotomy is not as clear in visceral infection of mice and even less in human visceral leishmaniasis [33]. The immune response and pathology of visceral leishmaniasis are complex, involving a number of genetic and cellular factors in the process of susceptibility or resistance to parasites [34]. 

### 2.1. Nonhealing Response

Susceptibility to visceral leishmaniasis is correlated with the presence of a Th2 response [33]. *L. donovani* infections stimulate expression of Th2 associated cytokines (IL-10, IL-4, IL-5, and IL-13). Further, elevated levels of IL-10 in serum and enhanced levels of IL-10 mRNA expression in lesion tissue are a direct indication of severe visceral leishmaniasis in mice and humans [35–38]. IL-10 plays a crucial role in the establishment and maintenance of Th2-dependent immune responses by suppressing Th1-dependent cell-mediated immunity in CL [39, 40]. In the murine VL model, IL-10 directly inhibits the antimicrobial machinery of macrophages by modulating normal signal transduction mechanisms [41]. IL-10 inhibits killing of amastigotes by downregulating the production of TNF-*α* and nitric oxide by macrophages and dendritic cells and thereby strongly impairs their antimicrobial activity [41, 42]. IL-10 also inhibits the production of IFN*γ*, one of the major proinflammatory cytokines [43]. However, in visceral leishmaniasis, it has been suggested that the impaired function of cellular immunity that correlates with progression of active disease may be due to the inhibitory effects of IL-10 independent of the IFN*γ* level [44]. 

IL-10, in addition, is also a regulatory cytokine mainly secreted by T cells, B cells, macrophages, dendritic cells, and keratinocytes [39, 45]. In experimental cutaneous leishmaniasis in mice, natural T regulatory cells (CD4+CD25+FoxP3+) are a major source of IL-10, and these cells are crucial for maintenance of parasite persistence [46]. However, in experimental VL in mice [47] and in human visceral leishmaniasis [37], IL-10 comes from non-T reg cells (CD4+CD25−FoxP3−), indicating that therapeutic approaches require *Leishmania* species-specific understanding of the immune response. IL-4 has generally been considered a Th2 cytokine that helps in the proliferation of the Th2 cell population and consequently a significant downregulator of Th1 cell response [48, 49]. Its role in CL is well defined but not in VL [32]. For example, the parasite burden of *L. donovani* in infected IL-4−/− and IL-4+/+ BALB/c mice was similar [50]. However, IL-4 null mice are more susceptible to visceral leishmaniasis than their wild-type counter parts after drug therapy [50], suggesting that IL-4 is necessary for effective chemotherapy in visceral leishmaniasis [50].

### 2.2. Healing Response

In VL (both in murine and human), resolution of infection depends on the production of Th1 cytokines [21, 31, 38, 51–54]. Production of IL-12 by antigen presenting cells and IFN*γ* by T cells are crucial for controlling the parasite growth and development of host immunity [51, 55]. There seems to be a dichotomy in the role of IL-10 in VL. On one side, IL-10 suppresses host immunity and helps parasite survival, on the flip side, IL-10 also protects the host from tissue damage by exaggerated inflammation [56]. Recent findings suggest that some IFN*γ* producing cells are a crucial source of IL-10, which act as a negative feedback mechanism to control tissue damage [57, 58]. A study with VL patient splenic T cells showed elevated expression of both IFN*γ* and IL-10 [37] suggesting that at least some cells are producing both cytokines as part of a strong inflammatory response and limiting the tissue damage by a feedback control mechanism [33]. TNF*α* also shares a significant role in resistance because TNF*α* −/**−** mice readily succumb to infection with *Leishmania* species [59, 60]. In addition, TNF*α* was also shown to stimulate the action of IFN*γ* in the induction of nitric oxide production in macrophages to kill the parasite [61]. Chemokines also play an important role in killing parasites from host cells [51, 62–64]. Antileishmanial activity of chemokines has been demonstrated in both *in vitro* and *in vivo* infections in murine VL by induction of respiratory burst as well as by induction of Th1 cytokines [65, 66]. Another CD4+ T-cell lineage called Th17 cells was initially characterized as producing IL-17 family cytokines [67]. They are proinflammatory cytokines that stimulate the production of IL-6, TNF*α*, and chemokines. A recent study suggests that *L. donovani* strongly induced IL-17 and IL-22 in human PBMCs and enhanced secretion of these cytokines correlated with protection [68]. 

In addition to CD4+ T cells, the significant role of CD8+ cytotoxic T cells in the control of experimental CL and VL has been described [69]. In murine experimental VL, control of parasite multiplication and leishmaniacidal activity are both associated with development of a granuloma in the liver, which requires both CD4 and CD8 T cells as well as IL-12, IFN*γ*, and IL-2 [30, 55, 70–73]. On the other hand, B cell deficient animals showing resistance to *Leishmania* infection and increased levels of IgG in human and canine VL (with few recent reports showing exceptions in these hosts) suggest that antibodies are not necessary for protection in the absence of an appropriate cellular response [22]. Thus, in addition to the protective T-cell response, the role of other T-cell subsets, including regulatory T and Th17 cells in either susceptibility or resistance to experimental and human visceral leishmaniasis, has not been ruled out and requires detailed investigations to help in vaccine designs.

## 3. The Need for a Genetically Altered Live-Attenuated Vaccine against VL

 Drug treatment requires long-term medication, which is expensive and highly toxic and often leads to resistance. Hence, vaccine strategies to combat leishmaniasis are very attractive. Though recombinant protein and DNA vaccines are under investigation, and in the past whole cell parasite lysates, heat killed parasites with or without adjuvants were used, a promising alternative is to develop live-attenuated parasite vaccines by genetic manipulation of genes that are necessary for virulence [22]. Moreover, live-attenuated pathogens have been used successfully as vaccine candidates against various viral and bacterial pathogens [19, 74]. In addition, the argument to use live-attenuated parasites as vaccine candidates becomes compelling based on the following facts: (a) complete *Leishmania *cDNA expression library injected into mice was more protective than any subpools of the library plasmids or a subunit, reinforcing the idea that the whole parasite makes the best vaccine [15]; (b) the cutaneous leishmanization practiced in the Middle East and Uzbekistan or the persistence of few parasites in the body even after cure protected individuals from reinfection [23]; (c) healing from natural or deliberate infection with *L. major* leads to the generation of more effective immunity against rechallenge in mice than vaccination with either killed parasites or defined leishmanial antigens that generally induce only short-term protection [75]; (d) in the past, researchers have developed attenuated strains by passaging through long-term culture, chemical mutagenesis, *γ*-irradiated, parasite culture under high drug pressure, and so forth [76–78]. Immunization with such attenuated strains showed a significant amount of protection upon virulent challenge in the mouse model, however, concerns about nonspecific attenuation that could result in reversion to infectivity of such strains does not make this a viable strategy. Further, nonspecific attenuation can lead to loss of protective immunity either by simply losing virulence or failure to establish subclinical infection. Therefore, the best alternate strategy would be to use “genetically defined live-attenuated” parasites. In the post genomic era with the help of *Leishmania* species-specific genomes, we can target a critical gene(s) that are responsible for survival inside macrophages, and deletion of such specific gene(s) by homologous recombination allows the selection of parasites lacking those critical genes for virulence. Further, we expect that immunization with such attenuated parasites will render protection against virulent infection by eliciting parasite-specific immune response in the host just like after recovering from natural infection. Most importantly, the live-attenuated organisms will have the complete antigen spectrum like their wild-type counterparts, which may result in a robust immunity as compared to subunit vaccines that cannot provide the complete repertoire of antigens. Finally, by its nature, the attenuated parasite will persist in the host for a certain period of time providing the immune system persistent antigens that may allow the generation of antigen-specific memory cells that can be mobilized to provide a protective response immediately following subsequent infection. All of these features put together make a compelling argument that the genetically defined attenuated parasite could be an alternative approach for a vaccine against leishmaniasis, including for VL. 

## 4. Development and Testing of Genetically Altered Live-Attenuated Vaccine Candidates against Leishmaniasis

 Several targeted gene deletions have been carried out to develop *Leishmania-*attenuated vaccine strains. Among the vaccine candidates developed for CL, *L. major *dihydrofolate reductase thymidylate synthase (*dhfr-ts-*) knockout parasites protect mice [79] but not rhesus monkeys [80]. *L. major *deficient in surface and secreted phosphoglycans (*lpg2−*), although unable to survive in sand flies and macrophages, retained the ability to persist indefinitely in mice and conferred protection against virulent challenge, even in the absence of a strong Th1 response [81, 82]. However, *lpg2−/−* parasites, over time, unexpectedly, regained virulence [83]. *L. major *deleted for phosphomannomutase (PMM) protected mice, despite no increase in either effector or memory response [84]. Additionally, *L. mexicana *that also causes CL, deficient in cysteine proteinase genes (Δ*cpa *and Δ*cpb*), conferred protection in mice and hamsters against homologous challenge [85, 86]. Among the vaccination studies in VL (Table 1), mice immunized with a *L. donovani *strain deleted for biopterin transporter (*BT1*) were also similarly protected from virulent challenge [87]. Recent attempts using partial knockout parasites for the A2-A2rel gene cluster in *L. donovani *[88] and SIR2 gene in *L. infantum *[89] as immunogens induced protection against virulent challenge in BALB/c mice. However, such mutants cannot be used as vaccine candidates, because they still carry wild type allele/s and could cause disease. There were also efforts to enhance the vaccine safety by including drug sensitive *L. major *mutants with suicide genes for controlled infection [90, 91]. Hence, these experiments, despite limitations with most of the mutant strains, demonstrate the potential as well as the pitfalls of generating live-attenuated vaccines by targeted gene disruptions. Hence, it is critical to develop attenuated lines through complete gene knockouts that generate organisms either with controlled infectivity that persists for a long time or parasites that persists for a brief duration and are eventually eliminated by the host immune system, thereby inducing effective immunity without clinical disease or the risk of reactivation. Towards this concept, we have developed several *L. donovani* mutant parasites, which lack virulence-specific genes. We developed a *L. donovani* mutant (*LdCen^−/−^*) deleted for the “centrin” gene. Centrin is a growth regulating gene in the protozoan parasites *Leishmania* [92], *Trypanosoma* [93] and *Plasmodium* [94], and higher eukaryotes [95]. *LdCen^−/−^* is specifically attenuated at the amastigote stage and not as the promastigote [96]. The mutant *Leishmania* amastigotes showed cytokinesis arrest in the cell cycle and persisted for a short duration in animals (mice and hamsters) or *ex vivo* in human macrophages and were eventually cleared [53]. In the animal studies, this attenuated parasite was found to be safe and protective in mice and hamsters against virulent challenge [53]. We also developed *L. donovani* mutant (Ldp27^−/−^) deleted for the “*p27*” gene. *Leishmania donovani *27 kDa mitochondrial inner membrane protein preferentially expressed in the amastigote stage is essential component of cytochrome c oxidase complex involved in oxidative phosphorylation [97]. The *Ldp27^−/−^* cell line is attenuated for infection of mice [97] and is currently being evaluated for its potential as a vaccine candidate. Taken together the examples of genetically altered parasites described above provide opportunities for live-attenuated vaccine candidates to be evaluated in pre-clinical and clinical conditions. However, there are various challenges to be addressed before such vaccine candidates become a reality. 

## 5. Challenges for the Use of Genetically Altered Live-Attenuated Parasites as Vaccine Candidates

### 5.1. Monitoring of Attenuation in the Live-Attenuated Vaccines to Ensure Safety

A main concern with live-attenuated vaccines is the risk of reversion to a virulent parasite, since these parasites are expanded to production levels and used as vaccines to inoculate recipients. Hence, biochemical or molecular indicators are needed to assess the genetic and physiological traits of the organism to assess stability of the attenuated parasites. Such indicators must be measurable with an assay that is practical in the manufacturing setting [99]. Therefore, in addition to monitoring the obvious presence or absence of the interested deleted gene, other genes, that are uniquely altered in expression in such gene deleted parasites, provide as indicators of attenuation. To date, there are no known defined indicators of attenuation for genetically altered parasites. Towards that end, our laboratory has begun to identify such biochemical or molecular indicators. For example, to assess genes whose expression patterns are indicators of attenuation, the *LdCen^−/−^* line was compared to wild-type by gene expression microarray [100, 101]. Two genes, one coding for the mitochondrial inner membrane protein (27 kDa protein) and another coding for putative Argininosuccinate Synthase, that normally express a higher RNA level in the amastigote stage than in the promastigote stage of wild type cells, were found downregulated in their RNA levels in *LdCen^−/−^* amastigote cells [99]. Northern blot analysis with these two genes showed that *LdCen^−/−^*parasites, recovered after five weeks of infection in mice, had the same expression pattern as they had prior to infection. Therefore, these two genes could be used as indicators of attenuation to monitor the safety of the *LdCen^−/−^* cell line as it is developed as a potential vaccine. However, further genetic evaluation to identify any compensatory mutations that could arise also will be an important aspect of any future such live vaccine candidate development.

### 5.2. Selection of Parasite Culture Conditions That Are Safe for Human Use

Live-attenuated parasite vaccines intended for injection into healthy individuals will need to be manufactured under strictly controlled conditions, that is, good manufacturing practices (cGMP) avoiding ingredients that could be hazardous for human use. One of the ingredients, bovine serum, that the parasite normally needs for its growth in culture, may not be suitable for human use. Even though in most cases the use of serum from BSE-negative animals is routine, there can be situations where it is not practical. Therefore, we have tested culture conditions for the genetically defined, attenuated parasites in serum free medium.

*LdCen^−/−^, Ldp27^−/−^*, and *Ld1S2D*, the parent wild-type strain of these genetically altered parasites, were adapted to growth in chemically defined medium (serum free media: SFM) [102]. SFM supported the growth of all tested *L. donovani* parasites at rates comparable with those obtained with serum-supplemented M199 medium (data not shown). Moreover, growth in such medium did not affect phenotypic characteristics of the parasites. We did not observe any difference in growth of mutant parasites compared to its wild type counterpart (data not shown). Parasite viability remained stable up to 6 days in culture (data not shown). Taken together, these results demonstrate the feasibility of cultivating *L. donovani *promastigotes in SFM and provide an alternative to use of serum for growing such parasites.

### 5.3. Vaccine Candidate Transmissibility by the Sand Fly

Another safety concern for genetically altered parasites to be used as vaccine candidates is whether such parasites, if taken up by sand flies, are able to survive in the sand fly gut and develop into infectious metacylic cells. The alterations made to date have been optimized for disrupting survival as amastigotes, yet allowing propagation as cultured promastigotes. Whether or not this will disrupt survival in sand flies cannot be predicted, but demonstration that the attenuated parasites do not survive or differentiate in the insect will be an important measure of safety blocking the potential for genetic recombination [103] or transmission of the vaccine strain. Preliminary studies from our laboratory suggest that genetically modified parasites may not be able to survive in the sand fly gut (Dey et al., unpublished data).

## 6. Immunoprotection against VL by Genetically Altered Live-Attenuated *Leishmania* Vaccines

In the literature, there are limited examples of genetically modified *L. donovani* parasites that have been tested against VL. The list of candidates is summarized in Table 1. Following are such examples. In our studies, mice immunized with *LdCen^−/−^* cells showed clearance of virulent challenge parasites in 10 weeks after challenge, with significantly reduced parasite burden in the spleen and no parasites in the liver. In contrast, high parasite loads were observed in the challenged mice previously immunized with heat-killed parasites or unimmunized [53]. Upon 10 week postvirulent challenge, the immunized mice displayed among the CD4+ T-cell population a significant increase of single and multiple Th1 type cytokine (IFN*γ*, IL-2, and TNF) producing T cells and increase in IFN*γ*/IL-10 ratio compared to nonimmunized naïve mice infected with wild type parasites. The naïve challenged mice displayed a reduced Th1 response and increased IL-10, a Th2 polarization accompanied by increased parasite burden in the organs. *LdCen^−/−^* immunized mice in addition showed increased IgG2a immunoglobulins and NO production compared to control [53]. These features indicated a protective Th1 response. Protection was observed, even when challenged 16 weeks after immunization with *LdCen^−/−^* parasites and in some cases even longer periods (24 week), signifying a sustained immunity and suggesting generation of a memory response. Thus, the immunity may remain after the absolute clearance of attenuated parasites demanded of a safe vaccine candidate. Protection by immunization with *LdCen^−/−^* parasites was also seen in hamsters [53]. In addition, we found that immunization with these mutant cells also cross-protected mice against challenge with *L. braziliensis* that causes mucocutaneous leishmaniasis (MCL) [53] and against *L. mexicana* that causes cutaneous leishmaniasis (CL) (unpublished data), indicating *LdCen^−/−^* to be a safe and effective vaccine candidate against VL, MCL, and CL. We also have recently developed another live-attenuated *L. donovani* parasite that lacks a gene that is part of the cytochrome c oxidase complex and is necessary for oxidative phosphorylation [97]. Preliminary data suggest that this could also serve as a live-attenuated vaccine candidate similar to *LdCen^−/−^.*

Th1 polarization towards protection against VL was also observed in animals after vaccination with other live-attenuated parasites (Table 1). Elevated IFN*γ* to IL-10 ratio with increased NO level and increase in both type 1 and 2 IgG antibodies correlating with parasite elimination was observed after *L. infantum* challenge in mice immunized with SIR2^+/−^ *L. infantum* [89]. *BT1* null mutant *L. donovani* parasites upon immunization in mice produced significant amounts of IFN*γ* correlating with protection against virulent challenge [87]. Immunization of BALB/c mice with nonpathogenic strain of *Leishmania* (*L. tarentolae*) expressing *L. donovan*i A2 antigen resulted in protective Th1 type of protective immunity against *L. infantum* challenge [98]. Finally, safety and efficacy of the genetically altered *L. donovani* live-attenuated candidate vaccines need to be assessed in large animals as was done for other Leishmanias. 

Generation and preservation of the immunological memory seems to be the most important but least studied aspect of antiparasitic vaccine development. In CL, the role of memory cells has been well studied and suggests generation of both effector (EM) and central memory (CM) T cells during infection [75, 104]. After antigenic stimulation, a few pathogen-specific EM T cells become CM T cells, which live for longer periods of time and stay in secondary lymphoid organs. Upon antigenic restimulation, CM cells become EM T cells and mediate protection [105]. Mice immunized with live-attenuated *L. major *parasites (DHFR deleted parasite) showed significant protection upon virulent parasite challenge, even after 25 weeks of immunization (in the absence of gene deleted parasites) and was due to the presence of CM cells [104]. Similarly, mice immunized with *LdCen^−/−^* vaccine candidate, that does not survive beyond 5 weeks after injection in mice, but shows protection when mice are challenged even 24 weeks after immunization [53] suggests *LdCen^−/−^* parasites could be generating a memory response that could be recalled upon challenge as was shown by Zaph et al. [104] for *L. major*. On the contrary, it is possible that protection by *LdCen^−/−^* parasites is achieved by establishing a low-grade persistent infection not detectable by current methods. This can be addressed by immunosuppressing immunized mice and monitor for parasite reemergence.

## 7. Other Considerations for Evaluation of Genetically Altered Live-Attenuated Vaccine Candidates

### 7.1. Experimental Models to Test Vaccines

The relevance of animal models is to understand the immunopathogenesis and to design, formulate, and test *Leishmania* vaccines/drugs. The models are expected to mimic the pathology and immunology observed in humans following inoculation with *Leishmania*. Criteria for establishment of infection in the animals are (i) susceptibility to infection, (ii) the age of the animal, and (iii) mode of infection. For *in vivo* testing of DNA, protein, or live parasite vaccines for VL, many animals have served as hosts, namely, BALB/c mice and Syrian golden hamster as primary screens, dogs as secondary screen, and squirrel, vervet, and Indian langur monkeys as tertiary screens [106–108]. Among such animals, the mouse may not be an ideal model for VL because in most situations the animals clear infection from both liver and spleen. However, the mouse model is used for VL studies in order to study the immune responses in the initial pathogenicity period since there are abundant immunological reagents available. Hence for VL, hamsters and nonhuman primates are more valuable models because of their similarity to human physiology and disease. The almost total lack of immunological tools for these animals is a disadvantage and it is not clear to what extent development or lack of immune responses in such animals are comparable to human. Among the nonhuman primates, African green monkeys were used as model to test infectivity of *Leishmania,* namely, *L. major* [109–111], *L. aethiopica* [112], *L. donovani* [113], and *L. infantum* [113], both langur monkey and squirrel monkey with *L. donovani* [114, 115], and owl monkey and marmoset with *L. braziliensis* [116, 117]. Few live-attenuated vaccines have been tested so far in mice/hamsters (Table 1). To our knowledge, no testing of live-attenuated vaccines against VL in non-human primates has been reported. However, a few vaccine studies conducted in monkeys against CL will provide insights in our understanding of vaccine studies against VL. The *dhfr-ts- L. major*, although protecting mice against CL, did not protect rhesus monkeys due to lack of protective immune response [79, 80]. However, in an another study, rhesus monkeys previously exposed to *L. major*, upon reinfection with *L. major* metacyclic promastigotes, showed reduced lesion compared to infection in naïve monkeys, signifying the immune response to live cutaneous parasites [118]. Limitations on the use of the dog or a monkey model are stipulated by the limited availability of such vaccine candidates in* Leishmania* endemic areas. Hence, it is important to forge collaborations with experts in nonendemic areas to address such questions. 

Our recent studies [119] and studies by others [120–124], with PBMC from Indian patients of VL, indicated elevated serum levels of IFN*γ*, IL-10, and IL-6 during the active disease, while TNF, IL-2, and IL-4 levels were minimal. Direct infection of healthy human PBMC *ex vivo* with live *L. donovani* parasites revealed elevated expression of IL-4 and IFN*γ* in the host cells [125]. Similarly, IFN*γ* upregulation was noticed in the PBMCs from nonexposed dogs after cocultured with *L. chagasi* promastigotes *ex vivo *[126]. Additionally, exposure of human PBMCs to peptides from a *Leishmania* surface antigen produced T cells that recognized *Leishmania*-infected autologous macrophages [127]. Since ethical reasons do not permit testing vaccine candidates directly in humans, as a preclinical evaluation of vaccines, the *ex vivo* grown human cells can be infected with live parasite vaccines (e.g., *LdCen^−/−^*) for a brief period and immune responses (Th1) potentially correlating with protection can be analyzed. 

### 7.2. Route of Administration and Mode of Virulent Challenge for Genetically Altered Live-Attenuated Vaccines

Studies to date with genetically targeted live-attenuated visceral *Leishmania* have primarily delivered the vaccine parasites intravenously [53]. If the vaccines are to be developed for human use, more acceptable routes of injection such as intramuscular, subcutaneous, or intradermal must be optimized. The intradermal infection route has been studied extensively for cutaneous *Leishmania* [128]; visceralization of *L. donovani* has been demonstrated after intradermal injection [129] and is being further investigated by our laboratory and others as a route of attenuated parasite vaccine administration. Subcutaneous injection of *L. chagasi-*attenuated parasites has been tested but failed to protect [130], though the nature of the vaccine strain, not the route of injection may be the cause of protection failure. As the attenuated parasite vaccine candidates are evaluated in larger animal models, these more favorable routes of administration will be tested.

The efficacy of a vaccine of any type is finally judged by its ability to protect recipients from natural infection. For leishmaniasis, this is a sand fly bite that delivers approximately 1,000 metacyclic promastigotes in the context of sand fly saliva, which is a potent modulator of the infection process [131, 132]. The traditional challenge models for visceral leishmaniasis are intravenous injection in the BALB/c mouse and intra cardiac injection of the hamster utilizing millions of stationary phase cultured virulent promastigotes- or spleen-derived amastigotes. The metacyclogenesis in culture is only partial; so stationary phase promastigotes is an imperfect model of the natural infection, but ficoll gradient separation of metacyclics improves the model [53]. The advantage of these challenge models is that they are well characterized, the disease outcome in a naïve animal is documented, and comparison of a current vaccine under evaluation can be made to earlier vaccine candidates. These models can be improved by dose modulation. A sufficiently virulent *L. donovani *strain that is maintained by continuous passage in hamsters will produce a robust infection with as little as 10^5^ stationary promastigotes from the first culture passage after harvesting from a hamster spleen. Low dose needle injection intradermally in the ear is a challenge model much closer to the natural infection; however, the uncertainties of visceralization and the long delay to disease symptoms may not be worth overcoming, especially in the light of recent demonstration that vaccines that protect against needle challenge do not protect against infected sand fly bites [133]. Insectaries in which to perform *Leishmania* infected sand fly transmission to rodent models are not common and are challenging to maintain. However, the benefits of evaluating candidate vaccines for efficacy against the natural sand fly mode of transmission will vastly improve the chance of ultimate success of live-attenuated *Leishmania* vaccine candidates. Towards that end, we and others have initiated studies to develop visceralization animal models (mice and hamsters) upon challenge with infected sand flies, which could be evaluated for the efficacy of genetically altered live-attenuated *Leishmania *parasites.

## 8. Conclusions

Unlike most other pathogens, *Leishmania* is never fully cleared by the immune system, depriving researchers of any natural correlate of immunity to mimic in designing a preventive vaccine that seeks to achieve sterile immunity. With vaccine candidates being tested, despite having been made empirically, detailed understanding is needed about the mechanisms by which the vaccines stimulate protective immunity. A methodical understanding of protective immune responses and generation and maintenance of the immunological memory during *Leishmania* infection is needed for a sustained long-term protective response. While developing attenuated strains of *Leishmania*, focus is required towards obtaining attenuation selectively at the intracellular stage (amastigotes), while remaining nonattenuated as promastigotes to permit large scale cultivation for use as vaccines. In order to enhance the potential of live parasite vaccines, optimization of attenuation needs to be explored, probably via a combination of gene deletions by knocking out more than one gene in a single *Leishmania* strain. There is a need to develop broadly immunogenic vaccines that induce protection against a wide range of *Leishmania* strains that are causative agents of many different clinical forms of leishmaniasis. Lastly, safety of genetically altered parasites is of paramount importance if such vaccines are to be used in humans. Hence, studies are needed to develop indicators of safety for such vaccine candidates. Upon success, such approaches will be applicable to develop live-attenuated vaccine candidates for other intracellular pathogens like malaria and bacterial infections.

## Figures and Tables

**Table 1:** Genetically altered live-attenuated vaccine candidates against visceral leishmaniasis.

Parasite	Characterization of attenuation	Animal model	Results of Immunization	References
*L. donovani*	Biopterin transporter gene deleted parasite (BT1^−/−^)	BALB/c mice	Protective immunity, antigen-specific IFN*γ* secretion	[87]
*L. tarentolae*	Nonpathogenic strain expressing *L. donovani* A2 antigen	BALB/c mice	Protective immunity against *L. infantum* challenge, high IFN*γ*, low IL-5	[98]
*L. donovani*	Replication deficient centrin gene deleted (Cen^−/−^)	BALB/c mice, Syrian hamster	Protective immunity against *L. donovani *and* L. braziliensis* challenge. Increased IFN*γ*, IL-2, and TNF producing cells and IFN*γ*/IL-10 ratio	[53]
*L. infantum*	Silent information regulatory 2 single allele deletion (SIR2^+/−^)	BALB/c mice	Protective immunity, increased antigen-specific IFN*γ*/IL-10 ratio	[89]
*L. donovani*	Cytochrome c oxidase complex component *p27* gene deleted cell line (Ldp27^−/−^)	BALB/c mice	12-week survival in host, initial evidence of protective immunity	[97] and Dey, unpublished
